# A toolbox of machine learning software to support microbiome analysis

**DOI:** 10.3389/fmicb.2023.1250806

**Published:** 2023-11-22

**Authors:** Laura Judith Marcos-Zambrano, Víctor Manuel López-Molina, Burcu Bakir-Gungor, Marcus Frohme, Kanita Karaduzovic-Hadziabdic, Thomas Klammsteiner, Eliana Ibrahimi, Leo Lahti, Tatjana Loncar-Turukalo, Xhilda Dhamo, Andrea Simeon, Alina Nechyporenko, Gianvito Pio, Piotr Przymus, Alexia Sampri, Vladimir Trajkovik, Blanca Lacruz-Pleguezuelos, Oliver Aasmets, Ricardo Araujo, Ioannis Anagnostopoulos, Önder Aydemir, Magali Berland, M. Luz Calle, Michelangelo Ceci, Hatice Duman, Aycan Gündoğdu, Aki S. Havulinna, Kardokh Hama Najib Kaka Bra, Eglantina Kalluci, Sercan Karav, Daniel Lode, Marta B. Lopes, Patrick May, Bram Nap, Miroslava Nedyalkova, Inês Paciência, Lejla Pasic, Meritxell Pujolassos, Rajesh Shigdel, Antonio Susín, Ines Thiele, Ciprian-Octavian Truică, Paul Wilmes, Ercument Yilmaz, Malik Yousef, Marcus Joakim Claesson, Jaak Truu, Enrique Carrillo de Santa Pau

**Affiliations:** ^1^Computational Biology Group, Precision Nutrition and Cancer Research Program, IMDEA Food Institute, Madrid, Spain; ^2^Department of Computer Engineering, Abdullah Gül University, Kayseri, Türkiye; ^3^Division Molecular Biotechnology and Functional Genomics, Technical University of Applied Sciences Wildau, Wildau, Germany; ^4^Faculty of Engineering and Natural Sciences, International University of Sarajevo, Sarajevo, Bosnia and Herzegovina; ^5^Department of Microbiology and Department of Ecology, University of Innsbruck, Innsbruck, Austria; ^6^Department of Biology, University of Tirana, Tirana, Albania; ^7^Department of Computing, University of Turku, Turku, Finland; ^8^Faculty of Technical Sciences, University of Novi Sad, Novi Sad, Serbia; ^9^Department of Applied Mathematics, Faculty of Natural Sciences, University of Tirana, Tirana, Albania; ^10^BioSense Institute, University of Novi Sad, Novi Sad, Serbia; ^11^Department of Systems Engineering, Kharkiv National University of Radioelectronics, Kharkiv, Ukraine; ^12^Department of Computer Science, University of Bari Aldo Moro, Bari, Italy; ^13^Big Data Lab, National Interuniversity Consortium for Informatics, Rome, Italy; ^14^Faculty of Mathematics and Computer Science, Nicolaus Copernicus University, Toruń, Poland; ^15^Victor Phillip Dahdaleh Heart and Lung Research Institute, University of Cambridge, Cambridge, United Kingdom; ^16^Faculty of Computer Science and Engineering, Ss. Cyril and Methodius University, Skopje, North Macedonia; ^17^Institute of Genomics, Estonian Genome Centre, University of Tartu, Tartu, Estonia; ^18^Department of Biotechnology, Institute of Molecular and Cell Biology, University of Tartu, Tartu, Estonia; ^19^Nephrology and Infectious Diseases R & D Group, i3S—Instituto de Investigação e Inovação em Saúde; INEB—Instituto de Engenharia Biomédica, Universidade do Porto, Porto, Portugal; ^20^Department of Informatics, University of Piraeus, Piraeus, Greece; ^21^Computer Science and Biomedical Informatics Department, University of Thessaly, Lamia, Greece; ^22^Department of Electrical and Electronics Engineering, Karadeniz Technical University, Trabzon, Türkiye; ^23^INRAE, MetaGenoPolis, Université Paris-Saclay, Jouy-en-Josas, France; ^24^Faculty of Sciences, Technology and Engineering, University of Vic – Central University of Catalonia, Vic, Barcelona, Spain; ^25^IRIS-CC, Fundació Institut de Recerca i Innovació en Ciències de la Vida i la Salut a la Catalunya Central, Vic, Barcelona, Spain; ^26^Department of Molecular Biology and Genetics, Çanakkale Onsekiz Mart University, Çanakkale, Türkiye; ^27^Department of Microbiology and Clinical Microbiology, Faculty of Medicine, Erciyes University, Kayseri, Türkiye; ^28^Metagenomics Laboratory, Genome and Stem Cell Center (GenKök), Erciyes University, Kayseri, Türkiye; ^29^Finnish Institute for Health and Welfare - THL, Helsinki, Finland; ^30^Institute for Molecular Medicine Finland, FIMM-HiLIFE, Helsinki, Finland; ^31^Institute of Molecular and Cell Biology, University of Tartu, Tartu, Estonia; ^32^Department of Molecular Biology and Genetics, Çanakkale Onsekiz Mart University, Çanakkale, Türkiye; ^33^Department of Mathematics, Center for Mathematics and Applications (NOVA Math), NOVA School of Science and Technology, Caparica, Portugal; ^34^UNIDEMI, Department of Mechanical and Industrial Engineering, NOVA School of Science and Technology, Caparica, Portugal; ^35^Bioinformatics Core, Luxembourg Centre for Systems Biomedicine, University of Luxembourg, Esch-sur-Alzette, Luxembourg; ^36^School of Medicine, University of Galway, Galway, Ireland; ^37^Department of Inorganic Chemistry, Faculty of Chemistry and Pharmacy, University of Sofia, Sofia, Bulgaria; ^38^Center for Environmental and Respiratory Health Research (CERH), Research Unit of Population Health, University of Oulu, Oulu, Finland; ^39^Biocenter Oulu, University of Oulu, Oulu, Finland; ^40^Sarajevo Medical School, University Sarajevo School of Science and Technology, Sarajevo, Bosnia and Herzegovina; ^41^Department of Clinical Science, University of Bergen, Bergen, Norway; ^42^Mathematical Department, UPC-Barcelona Tech, Barcelona, Spain; ^43^APC Microbiome Ireland, University College Cork, Cork, Ireland; ^44^Computer Science and Engineering Department, Faculty of Automatic Control and Computers, National University of Science and Technology Politehnica, Bucharest, Romania; ^45^Systems Ecology Group, Luxembourg Centre for Systems Biomedicine, Esch-sur-Alzette, Luxembourg; ^46^Department of Life Sciences and Medicine, Faculty of Science, Technology and Medicine, University of Luxembourg, Belvaux, Luxembourg; ^47^Department of Computer Technologies, Karadeniz Technical University, Trabzon, Türkiye; ^48^Department of Information Systems, Zefat Academic College, Zefat, Israel; ^49^Galilee Digital Health Research Center (GDH), Zefat Academic College, Zefat, Israel; ^50^School of Microbiology, University College Cork, Cork, Ireland

**Keywords:** microbiome, machine learning, software, feature generation, feature analysis, data integration, microbial gene prediction, microbial metabolic modeling

## Abstract

The human microbiome has become an area of intense research due to its potential impact on human health. However, the analysis and interpretation of this data have proven to be challenging due to its complexity and high dimensionality. Machine learning (ML) algorithms can process vast amounts of data to uncover informative patterns and relationships within the data, even with limited prior knowledge. Therefore, there has been a rapid growth in the development of software specifically designed for the analysis and interpretation of microbiome data using ML techniques. These software incorporate a wide range of ML algorithms for clustering, classification, regression, or feature selection, to identify microbial patterns and relationships within the data and generate predictive models. This rapid development with a constant need for new developments and integration of new features require efforts into compile, catalog and classify these tools to create infrastructures and services with easy, transparent, and trustable standards. Here we review the state-of-the-art for ML tools applied in human microbiome studies, performed as part of the COST Action ML4Microbiome activities. This scoping review focuses on ML based software and framework resources currently available for the analysis of microbiome data in humans. The aim is to support microbiologists and biomedical scientists to go deeper into specialized resources that integrate ML techniques and facilitate future benchmarking to create standards for the analysis of microbiome data. The software resources are organized based on the type of analysis they were developed for and the ML techniques they implement. A description of each software with examples of usage is provided including comments about pitfalls and lacks in the usage of software based on ML methods in relation to microbiome data that need to be considered by developers and users. This review represents an extensive compilation to date, offering valuable insights and guidance for researchers interested in leveraging ML approaches for microbiome analysis.

## 1 Introduction

The great development during the last decades in high-throughput technologies has allowed outstanding advances in different areas of knowledge like genomics (The 1000 Genomes Project Consortium et al., 2015), epigenomics (Stunnenberg et al., 2016), biodiversity (Lewin et al., 2018) or diseases (Boycott et al., 2019; Zhang et al., 2019). Microbiology has been paramount/highly integral here, in particular due to the reduction of costs and easy access have led to the creation of large volumes of data. Keystone microbiome projects like the Human Microbiome Project (The Human Microbiome Project Consortium, 2012), and the American Gut Project (McDonald et al., 2018) have collected 16S rRNA gene sequences for more than 31,000 and 15,000 human microbiome samples, respectively (date: 08/05/2023), whereas other general microbiome sequencing data repositories like MGnify include more than 147,000 human samples (date: 08/05/2023). This enormous volume of data has allowed the application of machine learning (ML) techniques in human research to support the classification of microbial DNA sequences, microbiome-related stratification of subjects, and the inference of host phenotypes in disease prediction/severity (Goodswen et al., 2021; Marcos-Zambrano et al., 2021; Yadav and Chauhan, 2022). The technology can provide useful and hidden patterns of information from large, noisy complex data like the microbiome. However, a number of challenges in the application of ML techniques in microbiology need to be addressed in terms of data type and quality, model interpretability, high dimensionality, or standards in development and deployment of ML techniques that have been reviewed elsewhere (Goodswen et al., 2021; Moreno-Indias et al., 2021).

Microbiome data has a high level of individual variation and can be influenced by known and unknown host-related processes. Therefore, ML can typically detect informative and hidden patterns in the data that might be with limited prior knowledge of the system in question. These algorithms can be divided into different categories, including supervised, unsupervised, semi-supervised and reinforcement learning (Sarker, 2021), whereof supervised and unsupervised methods are the most applied in human microbiome studies (Ghannam and Techtmann, 2021; Goodswen et al., 2021; Marcos-Zambrano et al., 2021). Previous work by the COST (European Cooperation in Science and Technology) Action CA18131 on *Statistical and Machine Learning Techniques in Human Microbiome Studies* (ML4Microbiome) has outlined the existing ML algorithms relevant for microbiome analysis (Marcos-Zambrano et al., 2021).

The complexity of microbiome interactions with the host, health outcomes, and the environment can be approached with the integration of different ML techniques and the exponentially growing body of microbiome data for a wide variety of applications in humans (Marcos-Zambrano et al., 2021). This is leading to the development of a wide array of specific software and frameworks that integrate different ML methods considering the different typologies of microbiome data. Microbiologists and biomedical scientists have a huge collection of tools to get the most out of their microbiome data, however, these tools are fragmented and dispersed among different repositories and publications. Frameworks for ML methods do not cover all different steps for microbiome analysis and the user often needs to combine different methods into a data science workflow to complete the analysis. Therefore, selecting the software and tools for microbiome data analysis requires diving into multiple repositories and resources being a time-consuming task at the rate at which these developments are growing in recent years.

Here, our aim is to go beyond the application of ML techniques in the microbiome field, extensively reviewed in the last few years (Ghannam and Techtmann, 2021; Goodswen et al., 2021; Marcos-Zambrano et al., 2021), and focus on a scoping review of ML-based software and framework resources currently available for the analysis of microbiome data in human studies. A description of each software with examples of usage is provided including comments about pitfalls and lacks in the application of ML methods in relation to microbiome data that need to be considered in software development. For a better understanding, the different pieces of software are organized by the type of analysis for which they were developed and the ML methods implemented. As far as we know, this is the most extensive catalog to date that intends to help microbiologists and biomedical scientists who are starting or wish to go deeper into specialized resources that integrate ML techniques for the analysis of microbiome data.

### 1.1 Specific software for ML applications in microbiome studies

In Supplementary Table 1 we summarize the most commonly used ML software for microbiome data analysis including the applicability (one application or more), availability of source code, last version, number of citations based on the Scopus database (this gives an idea about the level of usage), type of tool (level of deployment) and availability (public/commercial) for all the software and tools included. Each publication has been associated with the URL (pointed in the text) to the software described therein.^1^ Next, the software was evaluated in terms of the technologies used and the main ML tasks performed by the software. This allowed us to verify the most common ML tasks, the technologies used, and the change in the technologies used in recent years.

**Figure 1:**
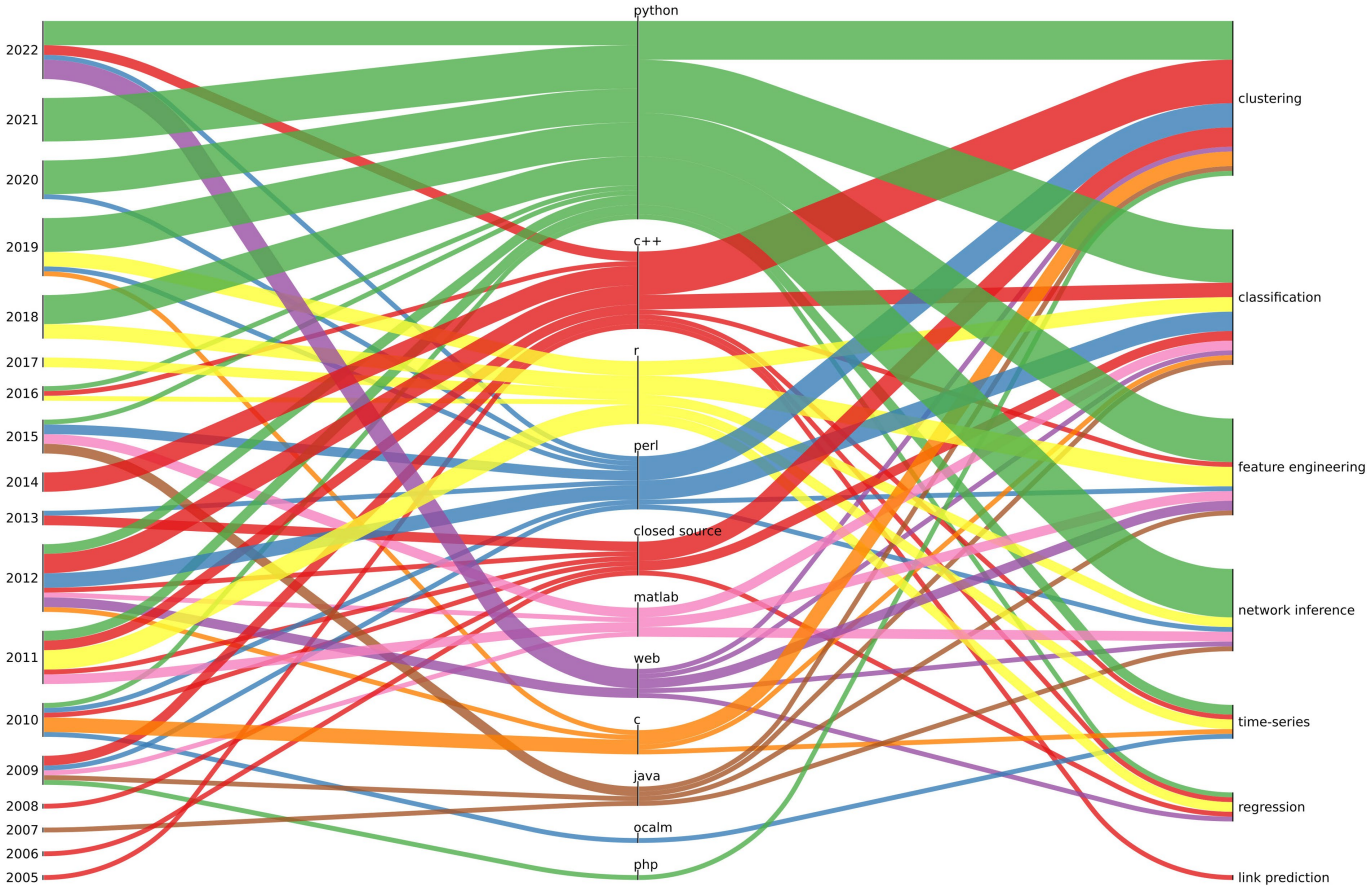
The relationship between the year of publication (left), programming lenguage (centre), and ML task (right) is depicted for the most commonly used software in microbiome analysis. The thickness of the line represents the quantity of software projects associated with a particular relationship (a project may have multiple relationships of given kind i.e., a software may be written in C and Python).

In Figure 1, we summarize the typical software stack used for microbiome tools over the years for given ML tasks. The thickness of the line indicates the number of publications divided into “year” - “programming language” and “programming language” - “ML task.” In recent years there has been a significant increase in the popularity of solutions created in interpreted programming languages (mainly Python and R) in relation to compiled programming languages (such as C/C++ or Java). With the exception of solutions written in the Perl interpreter, which has lost its popularity significantly over the years. There is a growing number of solutions using tensorflow for deep learning in microbiome research.

It should be noted that tool authors moved away from publishing software only in compiled (closed source) form (this trend could be observed until 2013 in our data), as closed source distribution of scientific software made verification impossible and contradicted the ideas of open science.

The last remark concerns the availability of the software after years, most likely due to the academic funding and career structure. Our observations show that as much as 11.5% of projects created between 2005 and 2022 are no longer maintained^2^ - and the software can only be found in the Internet Web archive.

**Figure 2:**
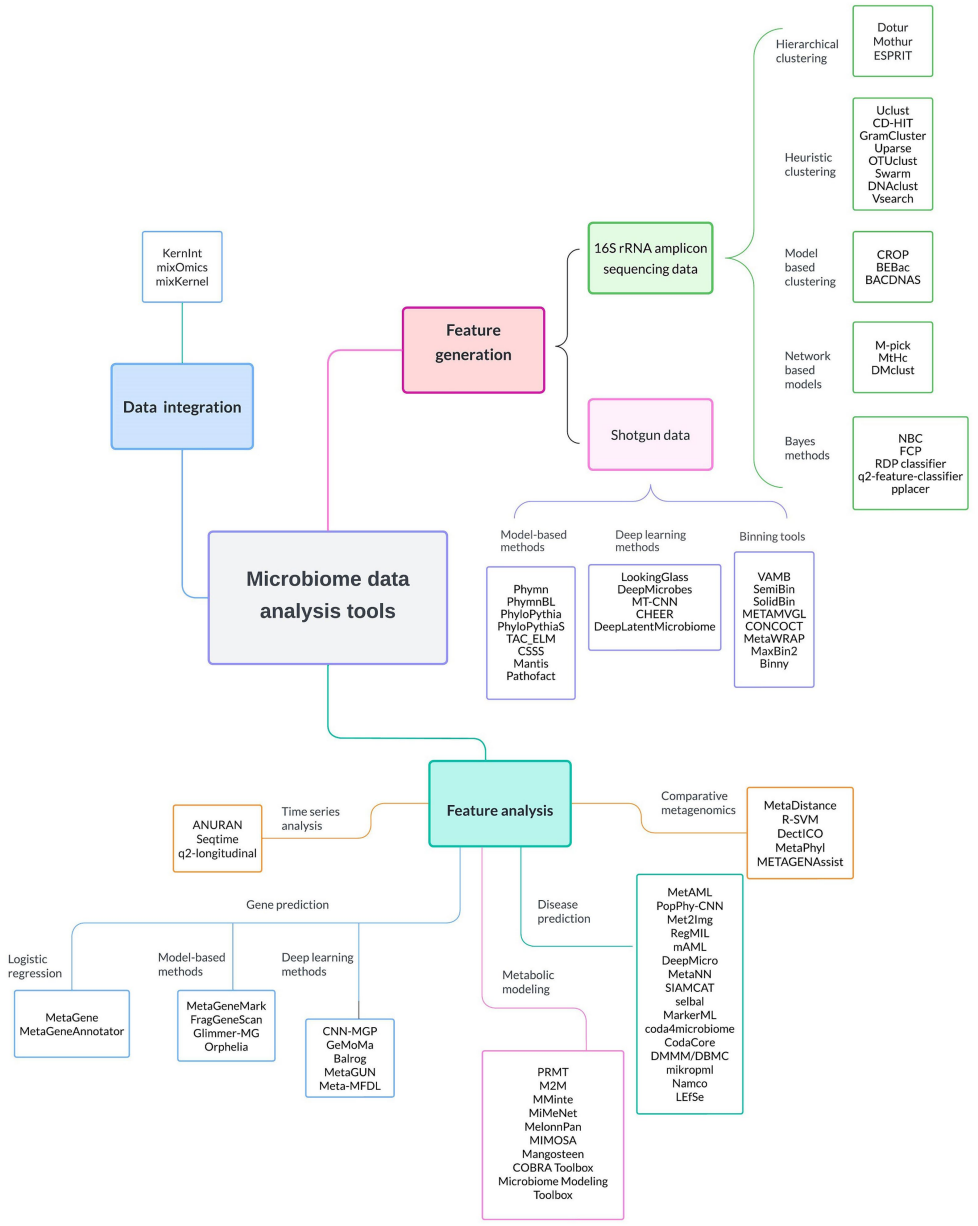
Comprehensive overview of the most commonly ML-based software applications employed in microbiome data analysis. These software tools are categorized based on their primary application into feature generation, feature analysis, and data integration. It is worth noting that numerous software options are applicable to both 16S rRNA gene sequencing data and shotgun metagenomics. Detailed descriptions of these software tools can be found in subsequent sections of the manuscript.

In Figure 2 we present a series of specialized ML software and tools used to facilitate several microbiome research steps. These steps include feature generation, where raw 16 s rRNA and shotgun sequencing data are processed and transformed into interpretable microbial units; data integration, where disparate datasets are combined for comprehensive analysis; and feature analysis, where a variety of tools are employed to perform time series analysis, gene prediction, metabolic modeling, disease prediction, and comparative metagenomics. These software and tools, discussed in detail in the next sections, can empower researchers to uncover the intricate dynamics within microbiomes and advance their understanding of their roles in human health. The emphasis is on ML software, and hence quite a number of very popular software in microbiome studies (Metaphlan, KneadData, and Kraken2,) would not be mentioned, due to omitting ML approaches.

Furthermore, we provide a comprehensive interactive table in the Supplementary materials that summarizes available software and tools for analyzing different types of microbiome data, organized according to their primary application (code accessible at https://github.com/laurichi13/Toolbox-ML-software).

## 2 ML-software for feature generation

In microbiome analysis features are usually generated by using two learning approaches: clustering and classification. Clustering is an unsupervised approach (an approach without a teacher) where the system forms groups of inputs (or clusters) according to the explicit or implicit rule and given a particular set of patterns or cost function (Duda et al., 2001). On the other hand, classification involves learning from a set of patterns whose category is known (i.e., supervised approach) and applying it to a set of patterns with unknown category, without any grouping.

### 2.1 Feature generation and taxonomic assignment from 16S rRNA gene sequencing

Human (and environmental) microbial analyses are often performed using 16S rRNA gene sequencing. This is possible as the 16S rRNA gene is highly conserved and universally present across prokaryotes. The 16S rRNA gene analysis implies using primers to amplify the hypervariable regions of the 16S rRNA gene (ranging from V1 to V9; frequently targeted for bacteria are the V3, V4, and V3-V4 regions; Nguyen et al., 2016).

Amplicon Sequence Variants (ASVs) provide a precise resolution of sequence variations without imposing arbitrary dissimilarity limits, unlike Operational Taxonomic Units (OTUs), which are commonly used in 16S rRNA data processing (Eren et al., 2013). ASV techniques utilize Illumina-scale amplicon data and can identify sequence differences as small as one nucleotide. They infer the biological sequences in the sample while considering amplification and sequencing errors (Callahan et al., 2017). On the other hand, OTUs cluster sequences based on similarity and assign representative sequences to proxy microbial taxa (Westcott et al., 2017; Wei et al., 2021).

### 2.2 Clustering of sequences (reads) for OTU/ASV assignment

Several clustering methods have been proposed, and several reviews are available with a solid methodological overview, limitations, performance comparison, and guidance in the selection of an appropriate clustering algorithm (Chen et al., 2013; Nguyen et al., 2016; Wei et al., 2021). Without the intention to provide a thorough evaluation of different OTU clustering methods, we here provide available tools for the generation of OTU tables, aiming to indicate the advantages and limitations of clustering approaches and resulting OTU features in general.

In contrast to the clustering-based OTU approach, the generation of ASVs can be described as a denoising method (Chiarello et al., 2022), where the algorithm gathers exact sequence variants *de novo* with little room for mismatches and determines their abundance. Based on the inferred ASVs, an error model is calculated for the dataset to compare highly similar reads in order to statistically exclude sequencing errors. This is based on the assumption that true biological sequences occur in higher frequencies than sequences emerging from sequencing errors. Moreover, unlike *de novo* clustered OTU, the identity of an ASV keeps its validity outside of the data set from which it was derived, thereby also simplifying meta-analyses of multiple data sets (Callahan et al., 2017). However, some limitations inherent to OTU-based methods such as multiple copies of the target region within an organism (e.g., 16S rRNA gene copy numbers) and the restricted information content of short reads also apply to ASV-based methods and should be considered in the interpretation of results.

#### 2.2.1 Hierarchical clustering

Creating clusters of data with similar characteristics is an approach to finding structure in data. Hierarchical clustering is an unsupervised learning technique for grouping similar objects into clusters. It creates a hierarchy of clusters based on similarity features within the data. Hierarchical clustering can be divided into two types: agglomerative (bottom-up) and divisive (top-down). The dendrogram construction depends on the type of linkage (i.e., the definition of distance between the clusters) used. The typical choices for OTU clustering are single linkage (which calculates the distance between the two closest objects belonging to each cluster, or nearest neighbor), complete linkage (which in turn is based on the distance between the two most distant objects, or furthest neighbor) and average linkage (unweighted-pair group), which is a compromise between the nearest neighbor logic of single linkage (Zhang et al., 2013). Once a hierarchical tree is constructed, the meaningful clusters can be defined by cutting the tree at a user-specified similarity threshold and merging all the sequences with higher similarity in the same OTU. Among these methods, the most familiar ones are Dotur (Schloss and Handelsman, 2005), based on Multiple Sequence Alignments, Mothur (Schloss et al., 2009), based on Needleman-Wunsch alignments against a pre-aligned reference database and ESPRIT (Sun et al., 2009), which implements a complete-linkage hierarchical clustering and minimizes the memory usage by adopting a k-mer distance for faster identification of very similar sequence pairs, producing sparse distance matrix. In hierarchical approaches, the number of sequences to be compared (N) determines the computational complexity [O(N^2^)], which usually renders these approaches more intensive as stated by the authors.

#### 2.2.2 Heuristic clustering of sequences

Heuristic clustering attempts to improve speed and scalability, avoiding exhaustive pairwise distance computation, and using a greedy strategy to form clusters based on an initial set of cluster seeds (Wei et al., 2021). Given a set of sequences, a subsequence is selected as a seed of a new OTU cluster. This subsequence is then compared to all remaining sequences of the given set of sequences. All sequences at the distance below the threshold with respect to any of the seeds are added to the corresponding OTU and removed from the sequence set. If no similar seed is found, a new cluster seed is formed from the query sequence. The performance of these methods is as well related to the selection of seeds. Some representative examples are Uclust (Edgar, 2010) and CD-HIT (Li et al., 2001; Li and Godzik, 2006). GramCluster (Russell et al., 2010) indexes the input dataset by a suffix tree for efficiency. Uparse (Edgar, 2013), an improvement of USEARCH (Edgar, 2010) and OTUCLUST (Albanese et al., 2015) rely on high quality sequences only, including steps for quality filtering, trimming, and chimera filtering. Swarm (Mahé et al., 2014) uses an agglomerative, unsupervised, single-linkage clustering algorithm that avoids the use of a global threshold. Each amplicon can be seen as a point in the discrete amplicon space, where its nearest neighbours have one nucleotide difference. User set parameter d is considered a tolerable similarity threshold, so that d-neighbours in the amplicon space are all amplicons with d nucleotide differences. Clustering amplicons starts from a seed, collecting all of its d-neighbours, and continues iteratively from these subseeds until natural cluster limits are reached, where no d-neighbours of any subseed can be added. In such a discrete amplicon space, amplicon clusters (OTUs) should be clearly separated contiguous regions, and the procedures ensures that all similar amplicons (i.e., amplicons close in the space) belong to the same cluster. DNACLUST (Ghodsi et al., 2011) adopts a greedy approach but improves the speed using filtering based on k-mers. There is an open-source 64-bit program VSEARCH (Rognes et al., 2016) which can be used instead of USEARCH, for which the source code and 64-bit versions are not publicly available.

#### 2.2.3 Model-based clustering

These methods attempt to circumvent the overestimation of OTUs due to the limitations of choosing an *a priori* similarity threshold (Chroneos, 2010; Huse et al., 2010). Setting a (hard) similarity threshold value directly affects clustering process and the resulting sequences’ partition, while using the probabilistic distance description fits better the nature of real data. The model-based methods, such as CROP (Hao et al., 2011) for example, tend to use Gaussian probabilistic distribution, indirectly targeting a certain similarity threshold, but being more flexible and thus more robust to sequencing errors and sequence variations. Moreover, the model based approaches imply very careful selection of model parameters, which is usually given as an optimization problem limiting the probabilistic parameter search to the parameter subspace in which the clustering results correspond to the desired partitions and to real number of OTUs (Hao et al., 2011). Other methods are BEBaC (Cheng et al., 2012), which is based on the calculation of an unnormalized posterior probability for an arbitrary partition of the reads, and BACDNAS (Jääskinen et al., 2014), which models sequences by Markov chains.

#### 2.2.4 Network-based models

They start from a graph construction which requires a full distance matrix between sequences, which involves computational burden, both memory and time consumption. Given this distance matrix, a weighted network is constructed and then a graph-based clustering method, based on the modularity community detection method, can be used for OTU picking (Wei et al., 2021). Some representative methods are: M-pick (Wang et al., 2013), MtHc (Wei and Zhang, 2015), and DMclust (Wei et al., 2017).

All of the clustering methods rely on similarity metrics and similarity thresholds used, which impact the output and quality of clustering. The selection of similarity measures is crucial, and research evidence indicates lots of criticism towards using percent sequence similarity in the OTU picking process (White et al., 2010; Schloss and Westcott, 2011). The reader is referred to Nguyen et al. (2016) for more insight into the problems of using sequence similarity for defining OTUs, which analyzes results obtained using three different dissimilarity metrics.

### 2.3 Taxonomic assignment of OTU/ASV

The procedures mentioned above for OTU/ASV clustering do not focus on species that constitute a sample. This is the goal of diversity profiling and taxonomic assignment. Diversity profiling aims to investigate the microbial community structure by providing an abundance of different taxa. The taxonomic assignment focuses on knowing which taxon belongs to each read or assembled contig. We can find two main kinds of software concerning these objectives: Naïve Bayes and Bayesian methods.

#### 2.3.1 Bayesian methods

The RDP classifier (Wang et al., 2007; Cole et al., 2009) relies on a reference sequence database that contains relevant species, and then assigns a class label to each read by the naïve Bayesian algorithm based on k-mer occurrence. Moreover, we can find NBC (Rosen et al., 2011) and the classifier FCP (Parks et al., 2011), which also implement a naïve Bayesian framework. pplacer (Matsen et al., 2010), is a software package for phylogenetic placement and subsequent visualization, which offers a full probabilistic and Bayesian framework to locate a query sentence in a reference phylogeny so that a taxon identifier can be assigned to the query sequence.

Through QIIME2 (Bolyen et al., 2019) plugin q2-feature-classifier (Bokulich et al., 2018a), it is now also possible to train an almost arbitrary classifier from the Python library Scikit-learn and use it to predict the taxonomy. The real shift in taxonomic assignment came with (Kaehler et al., 2019), when the increase in the species-level classification accuracy is achieved by incorporating environment-specific taxonomic abundance information. Classifiers for amplicon sequences, like Naive Bayes, assume that all species in the reference database are equally likely to be observed (Kaehler et al., 2019). However, in practice, the equal probabilities (or the uniform weights) assumption is not fulfilled resulting in reduced accuracy. As the authors explain (Kaehler et al., 2019), the accuracy is less if weight distribution is closer to uniform than if it is further. In QIIME2 it is implemented as a preprocessing step through its plugin q2-clawback. The plugin is used for assembling taxonomic weights, which are further used as input into taxonomic classification.

There are a few analysis methods for microbiome amplicon data that analyze the obtained data without having to pre-process the raw reads generated by sequencing to create feature tables of ASVs. Read2Pheno is a deep learning framework to predict phenotype from all the reads in a set of biological samples (Zhao et al., 2021). The software performs alignment-free microbial 16S rDNA sequence analysis to achieve read- and sample-level environmental prediction and extracts interesting sequence features using convolutional neural networks (CNN), recurrent neural networks, and attention mechanisms.

### 2.4 Feature table generation from microbiome shotgun sequencing data

In contrast to amplicon sequencing (e.g., of 16S rRNA genes), shotgun metagenomics involves sequencing of all or most microbial DNA in a sample. The DNA is cut into short fragments which are separately sequenced as compared to amplifying a particular genomic region, resulting in a large set of short DNA sequences (i.e., reads) that originates from different chromosomal regions from numerous genomes. Some of these reads are from genomic loci of taxonomic significance (like the 16S rRNA gene), while others are of coding sequences that reveal information about the biological processes encoded in the genome (Sharpton, 2014).

The analysis of metagenomic sequencing data involves numerous challenges. First, metagenomic data is relatively complex and large, rendering the processing more difficult. Furthermore, reads only partially reflect most genomes because most communities are too diverse. Because of the massive quantity of genomic information examined, metagenomic analysis typically requires a large volume of data to get relevant conclusions. This requirement may cause computing issues (both in terms of space and time). Fortunately, these algorithms are continuously advancing, making metagenomic analysis more accessible and efficient.

### 2.5 Taxonomic classification of short sequence reads

There are different types of ML methods used for the taxonomic classification of short sequence reads in metagenomic sequencing data. Model-based methods include Phymm and PhymmBL (Brady and Salzberg, 2009), which use interpolated Markov models to phylogenetically classify short sequence fragments. PhyloPythia and PhyloPythiaS (McHardy et al., 2007; Patil et al., 2012) use support vector machine classifiers based on k-mer frequencies to assign reads to pre-existing taxa. The CSSS method (Borozan et al., 2015) applies the nearest neighbor algorithm to assign taxonomic ranks to both bacterial and viral communities.

Deep learning models based on artificial neural networks that add several hidden layers and several neurons within each layer, are also used for taxonomic classification of short sequence reads in metagenomic sequencing data. These models are computationally expensive but often have high accuracy, and are good at capturing complex biological systems. TAC-ELM (Rasheed and Rangwala, 2012) is a composition-based method that uses a neural network-based model. LookingGlass (Hoarfrost et al., 2022) is a deep learning biological language model designed to capture the functional diversity of the microbial world by encoding contextually aware representations of short DNA reads. The model takes into account the order in which sequences appear and thus produces contextually relevant embeddings of biological sequences from microbial communities. Generated embeddings are able to differentiate sequences with different molecular functions, identify homologous sequences and differentiate sequences from disparate environmental contexts. Furthermore, LookingGlass may be fine-tuned by transfer learning to perform a variety of different tasks such as to identify novel oxidoreductases, to predict enzyme optimal temperature, and to recognize the reading frames of DNA sequence fragments. Liang and colleagues (Liang et al., 2020) developed a deep learning-based framework, DeepMicrobes, for taxonomic classification of short metagenomics sequencing reads that identifies potential uncultured species signatures in inflammatory bowel disease. This model achieved comparable accuracy in abundance estimation at the genus level when compared to state-of-the-art tools. The pipeline developed by Ma et al. (2021; MT-CNN) is based on a multi-task learning model that can perform both taxonomic assignment and estimation of genomic region for assigned reads for human viruses, together with a naïve Bayesian network which takes into consideration both the taxonomic assignments and the genomic coverage for the ranking of likely human viruses from sequence data. Ren et al. (2020) and Tampuu et al. (2019) proposed other deep learning-based approaches for classifying viruses from metagenomic reads. Shang and Sun (2021) presented CHEER, a tree-structure CNN pipeline for taxonomic classification of viral metagenomic data. PathoFact (de Nies et al., 2021) uses hidden Markov models and a random forest model in combination with the deep learning based DeepARG (Arango-Argoty et al., 2018) to predict virulence factors and antimicrobial resistance genes, while Mantis (Queirós et al., 2021) is a protein function annotation tool that uses database identifiers intersection and natural language processing based on text mining of protein function descriptions to integrate knowledge from multiple reference data sources into a single consensus-driven annotation.

### 2.6 Binning metagenome-assembled genomes

Binning is the computational process of assigning each read to a group called a bin, where each bin is expected to contain reads from the same taxon. Despite the existence of some alignment-based techniques (not covered in this review), the majority of computational tools for binning are currently in use in sequence *k*-mer composition. In fact, even when only dinucleotides (dimers) are taken into account, the distribution of k-mer composition is stable across a single genome and varies between genomes, as noted by Kariin and Burge (1995).

Binning is frequently used in environmental and human studies with the aim of establishing the taxonomic profile of a given sample. We distinguish between binning and taxonomic classification of amplicon sequences primarily based on the input data: whereas the latter is used in targeted studies, binning deals with assembled contigs from metagenomic reads from any genomic region of any sampled genome. Thus, binning is the method of choice for analyzing complex communities to determine near complete metagenome-assembled genomes (MAGs). However, almost all currently used techniques were created for bacterial communities, with MetaVir (Roux et al., 2011) being a notable exception as it focuses on the analysis of viromes. Other communities, like fungi, are frequently analyzed using *ad hoc* techniques or software tools intended for bacteria [see, for example, (Lindahl et al., 2013; Orellana, 2013)].

There are several binning tools available that use different methods as reviewed by Yang et al. (2021). For instance, VAMB (Nissen et al., 2021) uses deep learning in the form of variational autoencoders, while SemiBin (Pan et al., 2022) uses deep siamese neural networks in a semi-supervised approach. SolidBin (Wang et al., 2019) is based on semi-supervised spectral clustering, and METAMVGL (Zhang and Zhang, 2021) is a multi-view graph-based metagenomic contig binning algorithm. MetaDecoder (Liu C. -C. et al., 2022) is using a two-layer model based on Gaussian mixture models. Binny (Hickl et al., 2022) uses k-mer composition and coverage by metagenomic reads for iterative, nonlinear dimension reduction of genomic signatures as well as subsequent automated contig clustering with cluster assessment using lineage-specific marker gene sets. MaxBin2 (Wu et al., 2016) and CONCOCT (Alneberg et al., 2014) employ tetranucleotide frequencies (TNFs) and read depths to group together scaffolds. MaxBin2 utilizes an expectation–maximization algorithm to estimate the distances between scaffolds, while CONCOCT leverages Gaussian mixture models to cluster the scaffolds. However, there is no one-size-fits-all solution for metagenome binning, and ensemble-based tools like the binning module in MetaWRAP (Uritskiy et al., 2018) offer a promising approach to amalgamating binning results from various tools.

## 3 Analysis of features derived from amplicon or shotgun metagenomics:

### 3.1 Comparative metagenomics

This section includes techniques that label entire samples by examining features derived from each amplicon or shotgun DNA fragment from the sample (k-mers or OTU/ASV frequencies), sometimes supplemented with additional information (e.g., metadata, phylogenetics, class labels etc.). A common application of this classification in biomedical settings is phenotype analysis based on metagenomic fragments (Soueidan and Nikolski, 2016).

MetaPhyl (Tanaseichuk et al., 2014) is a two-phase heuristic algorithm for separating short paired-end reads from different genomes in a metagenomic dataset. The algorithm is based on the observation that most of the *l*-mers belong to unique genomes when *l* is sufficiently large. In the first stage of the algorithm, groups of l-mers are produced, each of which is associated with a single genome. Clusters are combined based on information from l-mer repeats during the second phase. Read assignments are made using these final clusters. The algorithm can handle very short reads and sequencing errors.

The study by Cui and Zhang (2013) employed R-SVM, which utilized generalized recursive Support vector machines (SVMs) to conduct feature selection and discrimination of human metagenome samples from control and inflammatory bowel disease patients. This alignment-free supervised classification approach can effectively differentiate between metagenomic samples belonging to predefined categories by selecting distinctive sequence features. The authors demonstrated the potential of utilizing metagenomic sequence features of microbiomes in the human body to investigate particular health conditions through supervised ML techniques.

DectICO (Ding et al., 2015) is a feature extraction, and dynamic selection-based supervised metagenomic classification method that can correctly classify metagenomic samples without relying on known microbial genomes and reads alignment. The tool combines SVM as the learning algorithm, intrinsic correlation of oligonucleotides (ICO), which generalizes the k-mer frequencies to describe samples, and kernel partial least squares for feature selection. When long k-mers are considered, the authors contend that DectICO performs better than other sequence-composition-based classification methods.

METAGENassist (Arndt et al., 2012) is a web server to make comparative metagenomics accessible to microbiologists. Users can upload their bacterial census, either amplified 16S rRNA data or shotgun metagenomic data, along with metadata (e.g., environmental, culture, and host conditions). All statistical analyses are performed by combining and normalizing user-submitted taxonomic profile data and automatically mapped phenotypic information (e.g., oxygen requirements, temperature range, habitat, host type, pathogenicity, disease association etc.) from METAGENassist’s phenotypic database. A variety of univariate methods are available for feature ranking regarding the significance of their changes due to the different conditions under study (e.g., fold change analysis, *t*-tests, Mann–Whitney tests, ANOVA, Kruskal–Wallis tests). Multivariate methods, namely, principal component analysis (PCA) and partial least squares discriminant analysis (PLS-DA), can be used for dimension reduction, visualization, classification, and feature identification. Hierarchical and partitional clustering methods are available to identify groups of samples regarding their feature abundance profiles, given their similarity based on a defined distance measure. For the prediction of attribute labels and the identification of important features (i.e., taxa or mapped phenotypes) METAGENAssist offers two methods, random forest and recursive SVM feature selection and sample classification (R-SVM). Mian (Jin et al., 2022) is another interactive web-based microbiome data table visualization and ML platform. Users can upload their metagenomic data as well as accompanying metadata, taxonomic mappings, phylogenetic tree or gene expression data. Mian allows users to preprocess their data, calculate alpha and beta diversity measures, apply feature selection methods and train ML models such as linear regressors, random forest or multilayer perceptrons. All tools are easy to tune and configure, and users will also be able to obtain common statistical measures as well as different plots for data visualization.

MetaDistance (Liu et al., 2011) is a MATLAB toolbox that comprehends the relationship between clinical phenotypes and microbiota profiles by developing new supervised learning tools. Instance-based [K-Nearest Neighbors (KNN)] and model-based (SVM) learning techniques have been combined to create the sparse distance learning approach (MetaDistance) that the authors have proposed for multi-class classification. The suggested approach is capable of class prediction and taxon identification in tandem. It can perform multi-class classification while not exacerbating any existing class imbalance. Additionally, this approach estimates only a few parameters, and specifically, the number of these parameters is equal to the number of features (input variables) in the dataset. This means that the model complexity is kept relatively low, which can be advantageous in scenarios with limited data or to prevent overfitting. It is very effective for metagenomic data issues, which frequently have small sample sizes, high dimensions, and unbalanced classifications with numerous classes.

### 3.2 Disease classification and feature prediction

The human microbiome is unique to each person and has been linked to various diseases, making it essential to associate the microbiome with the host’s disease state (Yadav and Chauhan, 2022). The disease status may be influenced by the presence of specific microbe species, their abundance, phylogenetic relationships, intermicrobial interactions, and microbial metabolites. ML models can be useful for this task because they account for the complex dependencies between microbial community members and can identify disease profiles and microbial biomarkers with limited prior knowledge. Abundance values of microorganisms, functional annotations of metagenomes, and k-mer abundances from raw reads are common features used for disease prediction (Bakir-Gungor et al., 2022). Microbial abundance profiles are commonly used as a feature in disease classification. This field is still in its early stages, and several ML approaches have been developed for classification based on disease-associated microbiome composition data (Bakir-Gungor et al., 2021). Here, we present several ML approaches designed for classification purposes given the disease-associated data about microbiome composition.

MetAML (Metagenomic prediction Analysis based on Machine Learning) is a computational tool for disease detection using gut metagenomic data. Here, SVMs, RFs, Lasso, Elastic Net, and other classifiers are implemented in this ML software framework for metagenome-based prediction tasks (Pasolli et al., 2016). Cross-validation allows for quantitative evaluation of model precision and adaptability to the general population. Evaluation metrics commonly used to measure the model’s performance include accuracy, sensitivity, specificity, precision, F1 score, AUC, among others (Table 1). MetAML has been tested on metagenomic case–control datasets from five different diseases, demonstrating potential for disease detection from gut metagenomic data. It has also been used in a study by Thomas et al. (2019), where ML models based on MetAML were developed to predict colorectal cancer using metagenome dataset. The models evaluated the prediction accuracies of the gut microbiome for colorectal cancer detection across populations and successfully identified consistent microbiome biomarkers and accurate disease-predictive models.

**Table 1:** Commonly used metrics to assess the performance and effectiveness of machine learning models.

Metric	Definition
Accuracy	Measures the overall correctness of the predictions made by a model. It is the ratio of the correctly predicted instances to the total number of instances in the dataset.
Sensitivity (Recall or true positive rate)	Quantifies the proportion of actual positive instances that are correctly identified as positive by the model. It is the ratio of true positive predictions to the sum of true positives and false negatives.
Specificity	Represents the ability of a model to identify negative instances correctly. It is the ratio of true negative predictions to the sum of true negatives and false positives.
Precision	Indicates the proportion of correctly predicted positive instances out of the total instances predicted as positive by the model.
F1 score	Is the harmonic mean of precision and sensitivity and provides a balanced evaluation of a model’s performance.
AUC (Area Under the ROC Curve)	The ROC curve plots the true positive rate against the false positive rate at various classification thresholds. AUC represents the area under this curve and is a measure of the model’s ability to discriminate between positive and negative instances.

PopPhy-CNN (Reiman et al., 2020) is a convolutional neural network (CNN) that predicts the host’s disease status using their microbiome samples. PopPhy-CNN involves transforming the phylogenetic tree and microbial abundance data into a structured matrix format. This matrix, enriched with evolutionary information, is then used as input for a CNN model to make predictions about the host’s disease status. The incorporation of biological knowledge through this process contributes to the model’s superior performance compared to other methods in binary classification and multi-class datasets. PopPhy-CNN models were more competitive than RF, SVMs, LASSO, 1D-CNN, MLPNN, and Ph-CNN models across nine moderately sized metagenomic datasets for binary classification (Qin et al., 2012, 2014; Karlsson et al., 2013; le Chatelier et al., 2013; Sokol et al., 2017). According to authors, PopPhy-CNN can deliver reliable performance with minimal training data and shows the best results for multi-class biological and synthetic datasets.

Met2Img (Hai Nguyen et al., 2019) is a disease prediction method that uses Synthetic Image Representations of Metagenomic data and CNN. The authors use a rectified linear unit (ReLU) activation function and transform each sample into an image containing coloured pixels representing the microbes and their relative quantities. The resulting images are subsequently used as features for the neural network. The authors evaluated the method using six metagenomic datasets, including five disease types and more than 1,000 samples. They reported encouraging results and held applicability across diverse omics data scenarios, including integrative contexts (i.e., taxonomic levels, CNN structure optimization, dimensionality reduction: effective colormaps, and GPU efficiency).

RegMIL is a Multiple Instance Learning (MIL) method that predicts phenotypes from metagenomic data. This approach employs a rapid, hash-based clustering technique referred as Canopy clustering to score instances in the training set. These scores estimate the contribution of an instance (sequence) to the disease. The instance scores of the training set are used to train a two-layer neural network-based regression model to score instances in the test set. In the end, one histogram-based bag-level feature representation by taking contributions of each instance to train a classifier (Rahman and Rangwala, 2018). RegMIL was shown to predict a person’s health status with high accuracy when evaluated with liver cirrhosis and IBS datasets, outperforming other tools like MetAML (Rahman and Rangwala, 2018).

mAML is an automated ML tool specifically designed for classification tasks performed on metagenomic data. The tool was developed in Python and the entire pipeline can be run through a web server, although it is also available to download and run locally. mAML preprocesses the data, performs grid-search for hyperparameter tuning, and provides several performance metrics for the classification task set by the user. The web-based tool allows the user to personalize each of these tasks. The mAML pipeline exhibits various benefits: (i) it can effectively and automatically construct an optimized, interpretable and resilient model for a microbiome-based classification task; (ii) it is implemented on a web- based platform (the mAML web server); (iii) the pipeline can be employed for both binary and multiclass classification tasks; (iv) it is data- driven and can readily be extended to encompass multi-omics data or other data types, given the availability of domain specific datasets (Yang and Zou, 2020). The authors evaluated mAML on 13 different metagenomic datasets, including binary and multi-class data. The models generated by mAML outperformed other models such as Support Vector Classifiers or logistic regression (Fierer et al., 2010; Wu et al., 2011; Qin et al., 2014; Montassier et al., 2016), demonstrating the method’s robustness. This method has been applied to predict carboxylate production from 16S rRNA gene dynamics (Liu B. et al., 2022).

DeepMicro is a deep learning method that is focused on the extraction of features from high dimensional microbiome data (more specifically extracted abundance and strain profile). It was shown to be more accurate than MetAML in transforming high-dimensional metagenomic data into a reliable low-dimensional representation for supervised or unsupervised learning (Curry et al., 2021). It was developed with disease prediction in mind, but has other applications. This approach could improve model performance for predictive problems using microbiome data, such as drug response prediction, forensic human identification, and food allergy prediction (Oh and Zhang, 2020).

DeepLatentMicrobiome which has an artificial neural network (ANN) architecture based on heterogeneous autoencoders (García-Jiménez et al., 2021), uses phenotypic features as well as environmental features (like temperature, precipitation, plant age, maize line and maize variety) to predict current or future microbiome compositions and can help scientists develop microbiome-engineering strategies with limited resources. Autoencoders are trained for each data source independently (thus acquiring heterogeneous autoencoders).

MetaNN (Lo and Marculescu, 2019) is a neural network-based technique that addresses challenges related to over-fitting and high dimensionality in metagenomic data, leading to improved classification accuracy. The method involves removing taxa that appear in less than 10% of the samples and generating additional samples using a negative binomial distribution to augment the training set. A neural network is then trained on the augmented dataset, resulting in superior performance compared to other ML models such as Random Forests, SVM and CNN, as demonstrated in evaluations by the authors Lo & Marculescu in 2019 using both synthetic and real datasets.

SIAMCAT is an R-based software that combines ML, statistical modeling, and advanced visualization approaches to enable comparative metagenomic studies. The tool provides normalization methods, cross-validation schemes, and implementation of various ML approaches such as LASSO (Tibshirani, 1996), Elastic Net (Zou and Hastie, 2005), and RF (Ho, 1995), among others. The trained models can then be used to make predictions based on the provided metagenomic data, and their performance can be measured using AUROC. According to Wirbel et al. (2021), SIAMCAT allows users to apply robust and verified ML models to their datasets, allowing pre-processing and normalization of the datasets depending on metagenomic data properties. It has been used in various studies, including those involving the classification of oral microbiome data (de Jesus et al., 2021) and the assessment of the association between microbiome composition and clinical responses to immune checkpoint inhibitor treatment (Lee et al., 2022). In the study developed by Kartal et al. (2022), it was discussed if fecal and salivary microbiota could be used as predictors of pancreatic ductal adenocarcinoma.

Namco is an R Shiny application designed for microbiome research that provides a wide range of data analysis tasks, including raw data processing, basic statistics (distribution of dominant taxa among groups), creation of heatmaps using different ordination methods, diversity analysis, network analysis, and ML (Dietrich et al., 2022). Among the latter, Namco offers users the ability to develop classification models using random forest to predict outcomes such as disease state or treatment response. The most important features in the classification are identified as biomarker candidates. The tool also enables time-series analysis and clustering to investigate microbial changes in response to treatment across different host development stages or over time.

LEfSe is a method for identifying metagenomic biomarkers that can explain differences between phenotypic classes. This method uses linear discriminant analysis (LDA) effect size (LEfSe; Segata et al., 2011). It is based on the non-parametric factorial Kruskal-Wallis sum-rank test to determine the statistical significance of differences found across classes. Biological consistency is then assessed using the Wilcoxon rank-sum test, and the effect size of each differentially abundant feature is estimated via LDA. Firstly, the Kruskal-Wallis test is employed to scrutinize all features and determine if there are dissimilarities in their distribution among different classes. Subsequently, features that contravene the null hypothesis undergo further analysis using the Wilcoxon test. This test compares all pairwise combinations between subclasses in different classes to ascertain if they conform to the general trend of the class. The resultant subset of vectors is then employed to establish an LDA model that ranks the features based on their relative differences among classes. Ultimately, the output is a list of discriminative features that are in line with the subclass grouping within classes and are ranked based on their effect size in distinguishing between classes.

MarkerML is a web server that employs interpretable ML and statistical testing to discover important metagenomic features (Nagpal et al., 2022). Its main goal is to identify marker-features, which can contrast comparable states and help in decision-making. Model interpretability is achieved by incorporating Shapley Additive exPlanations (SHAP)-based (Lundberg and Lee, 2017) analyses to detect predictive marker features. MarkerML also implements statistical testing methods to contextualize marker-feature discovery in metagenomic datasets, such as ANCOM-BC (Lin and Peddada, 2020; Lin et al., 2022) or ALDEx2 (Fernandes et al., 2013, 2014; Gloor et al., 2016). It also offers features such as access to databases (e.g., Taxonomic, KEGG, COG, PFAM), normalization options, feature selection, and multiple ML algorithms (e.g., XGBoost, Random Forests, Logistic Regression; Nagpal et al., 2022). MarkerML relies on class comparison and prediction for biomarker discovery, achieved by analyzing differential abundance and ML techniques, respectively.

Selbal is an algorithm whose objective is to find a microbial signature, i.e., a model defined by a group of microbial taxa whose pattern of abundance is predictive or associated with an outcome variable of interest (Rivera-Pinto et al., 2018). It uses the Selbal model selection method to find two groups of taxa whose relative abundance (referred as “balance”) sufficiently explains the target response variable (Rivera-Pinto et al., 2018). The algorithm iteratively runs multiple regressions while including a new taxon in the model each time. The two taxa whose balance is most closely connected to the response are the first ones that selbal selects. This approach has been used to differentiate between polycystic and non-polycystic ovary syndrome women (Lüll et al., 2021).

coda4microbiome (Calle et al., 2023) is an improved version of Selbal, which uses elastic-net penalization for joint variable selection in the all-pairs log-ratio model (i.e., the model that considers as explanatory variables all pairwise log-ratios of features). It outperforms Selbal by being more computationally efficient and allowing for different weights in the microbial signatures. While selbal uses forward selection, coda4microbiome applies elastic-net penalization on the “all-pairs log-ratio model” to perform joint variable selection. After reparameterization, the results are expressed as a microbial signature consisting of two taxa groups that are associated with the phenotype. coda4microbiome’s signatures are more versatile than selbal’s, as they allow different weights for taxa in each group, while selbal assigns the same weight to all taxa in each group. Coda4microbiome has also been implemented for both cross-sectional and longitudinal studies. The website of the project contains several tutorials.^3^ Other log-ratio based approaches for analyzing microbiome data include *CodaCore* (Gordon-Rodriguez et al., 2021) and the R package *amalgam* (Quinn and Erb, 2020), which aim to identify predictive balances or amalgams in a stepwise additive fashion. Some log-ratio based approaches in microbiome data analysis try to improve predictive accuracy by considering log-ratios that can contain several original features. However, many methods rely on pairwise log-ratios or additive log-ratios, which only involve two features. For example, the *easyCoda* R package includes three options for choosing pairwise log-ratios in a regression setting (Coenders and Greenacre, 2022), while the *logratiolasso* R package proposes a log-ratio LASSO model that aims to produce a sparse model from the all-pairs log-ratio model (Bates and Tibshirani, 2019).

DMMM/DBMC is a Dirichlet Multinomial Mixture Model (DMMM) tool that can be used in both unsupervised and supervised settings to identify clusters in microbiome datasets and act as a Bayes classifier. It is implemented in the R package *DirichletMultinomial* (Holmes et al., 2012) and was extended by Gao et al. (2017) to include automatic feature selection, resulting in better classification accuracy than DMMM and random forest.

mikropml is an R package that follows best practices for machine learning, producing trained models, performance metrics, and feature importances (Topçuoğlu et al., 2021). It includes data preprocessing, model training, and selection, as well as hyperparameter tuning. The package has been used to classify colorectal cancer patients and identify variables associated with bacterial infections (Topçuoğlu et al., 2021). The tool has also been applied to test ML models for associations between microbiome composition and diseases like *Clostridium difficile* infections, producing significant results in multiple studies (Lapp et al., 2021; Armour et al., 2022; Lesniak et al., 2022).

### 3.3 Gene prediction

Metagenomic studies aim to understand the metabolic and functional diversity of microbial communities and detect differences among them. However, establishing a complete geneset for each species in a sample is currently unfeasible. Gene prediction is a valuable tool in functional profiling, as it identifies patterns in DNA sequences that correspond to transcription and translation machinery. Here we present some of the most used algorithms including not-ML based prediction models.

Hidden Markov models (HMM) are commonly used in gene prediction, with several methods available. MetaGene (Noguchi et al., 2006) uses logistic regression models based on GC content and di-codon frequencies to differentiate between gene-coding and non-gene coding open reading frames (ORFs). MetaGeneAnnotator (Noguchi et al., 2008) extends this approach by integrating species-specific patterns of ribosome binding sites to improve translation start site prediction.

Model-based methods are commonly used in gene prediction, and there are several notable examples. MetaGeneMark (Zhu et al., 2010) is based on Hidden Markov models that are applicable to short DNA fragments. It uses training prokaryotic genomes to estimate polynomial and logistic approximations of oligonucleotide frequencies as a function of GC content. FragGeneScan (Rho et al., 2010) and Glimmer-MG (Kelley et al., 2012) both use Interpolated Markov Models to distinguish coding areas from non-coding DNA. Orphelia (Hoff et al., 2008, 2009) instead uses linear discriminants for mono-codon usage, di-codon usage, and translation initiation sites to extract characteristics from sequences, and also incorporates a neural network trained on random sub-sequences of genomes from the reference database to classify ORFs as protein-coding or not.

CNN-MGP (Al-Ajlan and El Allali, 2019) is a successful deep learning-based method for gene prediction. CNN-MGP avoids manual feature extraction and selection by predicting genes directly from raw DNA sequences. This method demonstrates the power of deep learning in accurate gene prediction. GeMoMa (Keilwagen et al., 2019) leverages evolutionary information from gene models in reference species to predict gene models in target species using amino acid sequence conservation, intron position conservation, and RNA-seq data. It is a homology-based gene prediction program.

Balrog (Bacterial Annotation by Learned Representation Of Genes; Sommer and Salzberg, 2021) is a model of prokaryotic genes based on a data-driven approach to gene finding with minimal hand-tuned heuristics. By training a single gene model on nearly all available high-quality prokaryotic gene data, this model matches the sensitivity of widely used gene finders.

ML-based methods have proven useful for metagenomic gene prediction. Meta-MFDL (Zhang et al., 2017) is a notable example that utilizes deep stacking networks to combine features such as monocodon usage, monoamino acid usage, ORF length coverage, and Z-curve features. This model has shown robustness and high accuracy in identifying metagenomic genes, outperforming other prediction models.

MetaGUN (Liu et al., 2013) is an ML-based method that uses SVM classifiers to identify protein-coding sequences in metagenomic fragments. MetaGUN uses entropy density profiles of codon usage, translation initiation site scores, and open reading frame length as input patterns.

### 3.4 Metabolic modeling

The metabolic activities carried out by the bacteria forming the gut microbiome are relevant for gut homeostasis and overall host health and physiology. These activities might not always be affected by taxonomic changes, and therefore it is essential to characterize microbiome-metabolome interactions. This will help to understand how shifts in the gut microbiome composition may affect host health, which in turn is crucial for the treatment and prevention of chronic diseases. In this section, we will describe methods that have been developed to characterize the metabolic activity of the microbiome.

Early modeling approaches focused on converting metagenomic features to metabolomic features due to the lack of comprehensive metabolomic profiles. The Predicted Relative Metabolic Turnover (PRMT) method (Larsen et al., 2011), originally developed for a marine metagenome, predicts metabolite consumption or production based on the enzymatic activities present in a metagenome. Briefly, it leverages information from KEGG and MG-RAST (reactions and EC numbers, respectively) to generate an environmental metabolomic matrix (EMM), estimates enzymatic activity based on number of sequences, and calculates a PRMT-score for each metabolite in the EMM (Larsen et al., 2011).

MIMOSA adapts this methodology in a multi-omic framework that combines taxonomic and metabolomic profiles in the context of the human microbiome (Noecker et al., 2016). This framework first infers community gene content based on taxonomic data and available and inferred genomic information. Then, making use of the PRMT method, it predicts the communitywide uptake or production of each metabolite, and estimates how species and genes might be contributing to these activities. Similarly to MIMOSA, Mangosteen is a metabolome prediction pipeline that relies on relationships between KEGG/BioCyc reactions and their associated molecular compounds (Yin et al., 2020).

However, with the increasing availability of both metagenomic and metabolomic data, numerous ML models have been developed to map metagenomic features to metabolites. These methods overcome the main limitation of reference-based methods, which are dependent on the quality of the queried databases. For instance, MelonnPan uses Elastic net regularization to predict community metabolomes from taxonomic profiles (Mallick et al., 2019). This approach has been used to predict metabolites in new microbial communities based on metagenomic data, shedding light on the functional role of microbiota in cardiovascular diseases (Liu et al., 2020).

Another ML-based approach, MiMeNet, is a multi-layer perceptron neural network that models microbe-metabolite relationships and the metabolomic profile of microbial communities from metagenomic taxonomic or functional features. This approach allows for scalability in handling large amounts of metagenomic and metabolomic features and leads to more robust predictive models by simultaneously learning metabolites and enhancing the transfer of information (Reiman et al., 2021).

Metage2Metabo (M2M) is another software tool that simulates the metabolism of the gut microbiota and describes the metabolic relationships between the species’ metabolic genes to establish how they complement each other in metabolic terms. M2M uses reference genomes or MAGs to construct genome-scale metabolic networks, which are then analyzed to detect metabolic capabilities and metabolic cooperation potential. Once this is carried out, M2M calculates the minimum number of species needed to perform a metabolic role of interest and the key species associated with that role (Belcour et al., 2020). M2M relies on the genome-scale metabolic network generating tool Pathway Tools (Karp et al., 2016).

Other approaches focus on constraint-based stoichiometric modeling using flux balance analysis (Orth et al., 2010) to determine the rate at which metabolites are being exchanged within the community (Thiele et al., 2013; Baldini et al., 2019; Heinken and Thiele, 2022). Constraint-based reconstruction and analysis (COBRA toolbox) is a software package for MATLAB, which allows for the creation and analysis of genome-scale metabolic models (Heirendt et al., 2019). It is reliant on the COBRA method which is a well-described set of strategies to employ when using metabolic modeling (Heirendt et al., 2019). Currently, the COBRA Toolbox is in its third edition and aims to simulate the relationship between genotype and phenotype through mathematical modeling (Heirendt et al., 2019). The Python COBRApy was developed as a framework allowing to model complex biological processes using COBRA methods (Ebrahim et al., 2013).

COBRA modeling has been used to create personalized human microbiome models and stratify them based on structure and function, which has been used to treat conditions such as inflammatory bowel disease and colorectal cancer (Heinken et al., 2021). It also supports other computational methods used for metabolome predictions with microbial data. For instance, MMinte (Mendes-Soares et al., 2016) relies on ModelSEED (Henry et al., 2010) and COBRApy (Ebrahim et al., 2013) for metabolic modeling and flux balance analysis (Mendes-Soares et al., 2016). This pipeline predicts metabolic interactions among microbial species in a community from 16S rRNA amplicon sequence data and association networks. It allows us to identify related genomes, reconstruct metabolic models, assess growth under specific metabolic conditions, analyze pairwise interactions, and generate a network of interactions (Mendes-Soares et al., 2016).

The COBRA method has also been used to construct organ-resolved whole-body human metabolic models, enabling simulations of both human and microbiome-human interactions (Heinken et al., 2020). In addition to the COBRA toolbox, the Microbiome Modeling Toolbox (Baldini et al., 2019) is a suite of MATLAB-based tools for building and analyzing microbe-microbe and personalized microbiome models. This toolbox generates, simulates, and interprets interactions between microbes and the host, as well as sample-specific microbial community models, using metagenomically derived data (Baldini et al., 2019). The updated version of the toolbox includes the mgPipe module, which facilitates the generation of personalized microbiome models from a vast collection of microbial metabolic reconstructions, such as the AGORA resource, containing over 7,000 microbial reconstructions (Magnúsdóttir et al., 2017; Heinken et al., 2020; Heinken and Thiele, 2022). The AGORA resource is also used by other tools, including the second version of MIMOSA (Noecker et al., 2016). Finally, MICOM is a customizable metabolic model of the human gut microbiome. Through COBRApy, it calculates growth rates based on metagenomic and dietary characteristics, allowing for the generation of personalized metabolic models for individual metagenomic samples (Diener et al., 2020).

### 3.5 Time-series analysis

Time-series data analysis is essential for understanding the structure and dynamics of microbial communities. However, it requires specialized statistical considerations distinct from those used in comparative microbiome studies to address ecological questions. To facilitate this, some software packages have been developed that use ML algorithms to analyze time-series data.

One such package is QIIME2 plugin q2-longitudinal (Bokulich et al., 2018b), designed for the analysis and visualization of longitudinal microbiome studies. This QIIME2 plugin incorporates various methods for paired difference and distance testing, linear mixed-effects models, nonparametric microbial interdependence, feature selection and volatility analysis, and interactive visualization. The feature-volatility action uses random forests to identify features that change over time and predict different states.

Another package is Seqtime, an R package that provides functions to analyze sequencing data time-series and simulate community dynamics (Faust et al., 2018). Additionally, the Anuran toolbox helps identify conserved or unique patterns across multiple networks over time, and whether biological networks have set operations that have different outcomes than expected by chance (Röttjers et al., 2021).

## 4 Data integration

The complexity and heterogeneity of the metagenomics datasets, which include various types, scales, and distributions, make it challenging to extract useful information from them in the context of omics data mining. One of the main obstacles to the successful use of ML techniques in metagenomics analysis is the integration of such a wide variety of heterogeneous data.

Picard et al. (2021) classified integration approaches into horizontal and vertical categories. Within the vertical integration strategies, further divisions include early, mixed, intermediate, late, and hierarchical approaches. Early and intermediate integration strategies enable the analysis of datasets within the context of their relationships with other datasets, leading to additional insights. However, early integration is challenging for most ML models, while intermediate integration often relies on unsupervised matrix factorization, which lacks the incorporation of pre-existing biological knowledge. Late integration involves applying ML models separately to each dataset and then combining their predictions. Hierarchical integration considers the interaction between different layers of omics data explicitly, but its implementation is currently in its early stages.

Most of the integration approaches implemented in software packages are based on mixed integration, which typically first modifies and transforms each dataset using different ML models. This enables them to reduce data complexity and heterogeneity, as well as to facilitate subsequent integration and analysis of datasets. Here we collect some of the ML software used for metagenomics data integration:

There are several software packages available for metagenomics data integration. mixOmics (Rohart et al., 2017a) for example, is an R package that provides a wide range of multivariate methods for data exploration, sizing, and visualization, including integration platforms that investigate relationships between heterogeneous omics data (in terms of types, scales and distributions). Its multivariate projection-based methods are computationally efficient for processing large omics datasets and provide flexibility in analyzing biological datasets by using relaxed assumptions about the distribution of the data. MixOmics R includes both supervised and unsupervised frameworks as well as feature selection. Other frameworks, like DIABLO (Singh et al., 2019) and MINT (Rohart et al., 2017b), enable the integration of datasets to identify relevant relationships and significant patterns in heterogeneous data for better exploration of complex metagenomic data.

Kernel methods allow data scientists to model non-linear relationships between the data points with low computational complexity, thanks to the so-called ‘kernel trick’. These have already been used to extend well-known algorithms such as PCA, linear DA and ridge regression (Cabassi and Kirk, 2020). A consensus multiple kernels is based on ideas similar to STATIS as an exploratory method designed to integrate multi-block datasets when the blocks are measured on the same samples (Mariette and Villa-Vialaneix, 2018). MixKernel (Mariette and Villa-Vialaneix, 2018) is another R package that offers methods for integrating heterogeneous types of data, focusing on kernel fusion methods for unsupervised exploratory analysis. Its kernel methods allow data scientists to model non-linear relationships between the data points with low computational complexity, thanks to the so-called kernel trick. KernInt (Ramon et al., 2021) is a kernel framework for integrating supervised and unsupervised analyses in spatiotemporal metagenomic datasets, using a kernel framework to unify supervised and unsupervised microbiome analyses, focusing on spatial and temporal integration, including the retrieval of microbial signatures.

### 4.1 General software for machine learning applications

A variety of ML software tools are available, with the majority being open source. Goodswen et al. (2021) and co-authors have compiled a brief list of general ML software tools to be applied in microbiome data. We have here extended this list in Supplementary Table 2 to include additional relevant general ML software for microbiology data analysis. These tools are primarily based on Python and R frameworks that contain collections of software libraries (packages) and require some basic programming knowledge for optimal use. However, some ML tools like WEKA, KNIME Analytics Platform, and Orange Data Mining, can be used through a GUI without extensive coding or programming expertise.

### 4.2 Commercial approaches and solutions

We identified more than 240 companies (in >350 locations) worldwide based on an online database of companies applying or offering microbiome analysis (Microbiome Employers, 2022) complemented with search engine results.

The companies’ activities ranged from clinical research and the study of diagnostic and therapeutic effects in healthcare to the implementation of microbiome data analysis in agriculture, nutritional supplements and pharmaceuticals. The majority of these address microbiome analysis for therapeutics/pharmacy. Three typical examples are the discovery of novel molecules for therapeutics, agriculture, and nutrition (Adapsyn Bioscience, 2022), the prediction of viable biomarkers and therapeutic candidates against immunologic disorders (Pragmabio, 2022) and microbiome tests as a diagnostic application in medicine and cosmetics (Atlas Biomed, 2022).

For obvious reasons not to disclose proprietary knowledge or internal processes, the companies were mostly not willing to disclose details on their use of ML. With that said, 60 companies do apply ML according to stated keywords like ‘Machine Learning’, ‘AI’, or ‘Deep Learning’ in a given context on their websites. More detailed information about the used algorithms were, however, normally not available. The companies offering microbiome analyses and integrating ML methods either do this as part of a sequencing service (e.g., CosmosID, www.cosmosid.com) or consider microbiome analyses as a part of a more thorough analysis. Good examples of the latter with a “microbiome-subsection” in their product portfolio are Ardigen^4^ with a precision medicine service or AstarteMedical^5^ with their digital tools and diagnostics to improve pediatric outcomes. A more general approach is followed by EagleGenomics^6^ which offers a platform-driven whole microbiome analysis ecosystem.

### 4.3 Challenges of ML to consider in software development for microbiome applications

#### 4.3.1 Bias and variance

Almost all ML approaches introduce some bias (Quinn, 2021) in the training phase, i.e., assumptions on the model “shape” and on the data distribution made during the construction of the model. When such assumptions hold, the model tends to be highly accurate, both in the training set and in the testing set, but when such assumptions are violated, such bias can lead the method to miss, ignore or discard relevant relations between descriptive features and the target feature. Approaches that exhibit a high bias can therefore lead to *underfitting*.

On the other hand, ML approaches can also generate variance errors, specifically, when they are very sensitive to small fluctuations in the training set. This issue can ultimately push the algorithm to specifically model the random noise present in the training data. When this occurs, the learned model is very accurate on the training set but poorly generalizable to the unseen data of the testing set (*overfitting*). These phenomena, in the specific context of microbiome data, have been recently emphasized in some papers (Lin and Peddada, 2020; Nearing et al., 2021; Wirbel et al., 2021).

It is noteworthy that the above-mentioned phenomena occur in almost all the application domains, not only when analyzing microbiome data, and the possible solutions tend to be common to those generally adopted in other contexts. However, since the first attempts at the adoption of ML approaches to microbiome data analysis are very recent, the context is probably not mature enough for the adoption of methods with a high bias. Solutions like multi-view learning, semi-supervised learning and transfer learning can be profitably used to alleviate such problems.

### 4.4 Impact of dataset size on the model accuracy

In general, the availability of large amounts of data in available repositories such as NCBI,^7^ METAHIT,^8^ Human Microbiome Project,^9^ ExperimentHub,^10^ etc., increases the chance of learning accurate ML models, and the impact of the dataset size on the model accuracy depends on the data source. However, it varies on the basis of the specific problem at hand. For example, fewer data are required if there are clear patterns within the data, if they are easily separable (in the case of classification tasks), or if simple (e.g., linear) relationships can be identified between descriptive and target attributes (in the case of regression tasks). In addition, some ML algorithms inherently require huge amounts of data due to their complexity (e.g., the number of parameters to optimize): simpler methods, such as linear regression and decision trees, typically need less training examples than solutions based on deep learning.

In microbiome research, the number of available samples is usually very limited due to sequencing costs and logistical challenges of sample collection. This aspect limits the adoption of complex approaches, although very promising according to the results achieved in other contexts. A possible solution to alleviate this issue would consist in relying on approaches that are able to exploit the knowledge coming from other contexts with huge amounts of labeled examples, such as transfer learning methods (Pio et al., 2022), or that can exploit both labeled and unlabeled examples (which may be less expensive to gather) in a semi-supervised learning setting (Chapelle et al., 2010), also based on multi-view learning (Ceci et al., 2015).

### 4.5 Data quality

Even when large data sets are available, there is no guarantee that the available data sample represents the whole population, without (selection or other kinds of) biases. In addition, available data sets may also include examples with (i) incorrect labels, (ii) missing or wrong values in the descriptive features, possibly due to measurement errors, (iii) highly dimensional and very sparse representation, due to the usual scarce availability of individuals with respect to the large availability of (also incomplete) generated features. The presence of one or more of such issues requires the adoption of pre-processing techniques. However, general-purpose methods may introduce additional noise or remove/discard relevant information, which suggests the need to focus on specific approaches for handling the peculiarities of microbiome data.

Another possible solution would consist in integrating multiple data sources, or in combining multiple pre-processing methods, in an ensemble or multi-view fashion. This is also confirmed by Curry et al. (2021), who states “A major source of future advancement in phenotype-prediction would be the result of discovering new data sources or feature types that have complementary predictive power, then utilizing the appropriate model structures for leveraging additional information.” This approach can turn out to be effective also in the case we use features generated using existing methods (such as OTU, ASV, Metagenome-profiling, etc.) since it provides an automatic and data-driven way to merge feature contributions.

## 5 Interpretability and explainability

The interpretability of the results of the analysis of microbiome data is a very difficult task (Feng et al., 2015; Yu et al., 2017). In order to support this activity, the ML community is recently giving attention to the problem of model interpretability, and explainability of the predictions. This is motivated by the fact that ML models are adopted in critical decision environments, like security, health and biology, which cannot generally accept a blind output of an automated system. The importance of such an issue has been perceived even more recently, due to the general spread of neural network architectures to solve several ML tasks, which are generally very accurate but inherently not interpretable. This issue is present also in the context of microbiome data (Carrieri et al., 2021), especially when they are adopted for diagnostics purposes. Therefore, together with the design and development of accurate ML methods, able to work with sparse, high-dimensional, and noisy data, the effort of the research community should focus on the design of methods able to learn explainable models, in order to generally increase their acceptance in the biomedical field.

## 6 Conclusion

ML techniques are powerful methods for analyzing the huge amount of data that is being generated in the human microbiome field (Marcos-Zambrano et al., 2021; Moreno-Indias et al., 2021). As discussed in this manuscript, its application is leading to a rapid growth of specific ML tools to support and facilitate the different steps in the analysis and interpretation of microbiome data. These software developments democratize access to ML techniques, making them more accessible and easier to use for a wide range of organizations and researchers. However, the shortcomings and challenges of the ML application in human data, reviewed extensively by the COST (European Cooperation in Science and Technology) Action CA18131 on *Statistical and Machine Learning Techniques in Human Microbiome Studies* (ML4Microbiome) in Marcos-Zambrano et al. (2021) and Moreno-Indias et al. (2021), along with the fragmentation and dispersion of the ML software and microbiome data require further efforts to create federated infrastructures and services, as stated by the European Open Science Cloud (European Commission Directorate General for Research and Innovation. and EOSC Executive Board, 2021) or ELIXIR (Balech et al., 2022), to exploit complex human microbiome data accelerating innovation, and ensuring that the benefits of ML are distributed more broadly across society, these tools can help drive progress and create a more equitable and sustainable future. Hence, ML4Microbiome contributes to this aim with a very valuable resource to microbiologists and biomedical scientists identifying and cataloguing the ML software available, facilitating and supporting the analysis and interpretation of large human microbiome datasets. This paper is part of a series of publications that emerged from the efforts of COST Action ML4 Microbiome. Other articles will address challenges (ID 1257002), data transformation (ID 1261889, ID 1250909), and best practices. The primary focus of this particular article is to gather and present a comprehensive range of ML resources and tools that are available for metagenomic analysis. In the future, benchmarking efforts by the community will be required to evaluate the performance, accessibility and user experience of these tools to provide non ML expert users with easy, transparent, and trustable standards. As the availability of methods and the vast number of workflow choices spanning unique combinations of preprocessing, feature selection, ML algorithm, parameterization, optimization, and other technical details often have remarkable effects on the analysis outcomes, the field benefits from independent benchmarking of alternative machine learning approaches. Independent competitions and community challenges provide one route for this. A recent example of this is the Heart Failure Prediction Microbiome FINRISK DREAM challenge (FINRISK, 2022), which was organized by the ML4microbiome COST action to identify optimal strategies for microbiome-based prospective risk prediction for heart failure using large-scale population cohort data sets and which results will be published soon. In addition, It will be required that software developers follow Findable, Accessible, Interoperable and Reusable (FAIR) principles for a more efficient use of resources, get more accurate results and better decision-making.

## Author contributions

LM-Z and EC: conceptualization, supervision, and writing – original draft. VL-M: investigation and writing – original draft. BB-G, MF, KK-H, TK, LL, TL-T, XD, ASi, AN, GP, ASa, and VT: investigation, validation, and writing – review and editing. EI and PP: visualization, investigation, and writing – review and editing, BL-P, OA, RA, IA, ÖA, MB, MC, HD, AG, AH, EK, SK, DL, ML, PM, BN, MN, IP, LP, MP, RS, ASu, IT, C-OT, PW, EY, MY, MC, and JT: investigation and writing – review and editing. MC, JT, and EC: funding acquisition. All authors contributed to the article and approved the submitted version.

## Footnotes

^1^Up to 11.5% of the URLs were pointing to non-existent or outdated pages - in this case, the link to the software was checked with the Internet Archive (https://web.archive.org) to find a page corresponding to the described software.

^2^The url provided in the publication to the software points to non-existent resources, and there is no redirection to a new page.

^3^https://malucalle.github.io/coda4microbiome/

^4^https://ardigen.com/

^5^https://astartemedical.com/

^6^www.eaglegenomics.com

^7^https://www.ncbi.nlm.nih.gov/

^8^https://www.gutmicrobiotaforhealth.com/metahit/

^9^https://hmpdacc.org/

^10^https://bioconductor.org/packages/release/bioc/html/ExperimentHub.html
