# Supplementary material for: A toolbox of machine learning software to support microbiome analysis
Source: Front Microbiol. 2023 Nov 22;14:1250806. doi: 10.3389/fmicb.2023.1250806 (PMC10704913; doi:10.3389/fmicb.2023.1250806)
Supplement: Supplementary file 1 [file Data_Sheet_1.ZIP › Toolbox-ML-software-main/Supplemntary_final.html]

A toolbox of machine learning software to support microbiome analysis


# A toolbox of machine learning software to support microbiome analysis

Analysis

All
Feature generation
Feature analysis

Data

All
16S rRNA amplicon sequencing data
Gene prediction
Shotgun data
Features

Method

All
Model-based clustering
Deep learning methods
Metagenome assembled genomes
Heuristic clustering
Metabolic modeling
Disease prediction
Model-based methods
Comparative metagenomics
Network-based models
Hierarchical clustering
Bayes methods
Binning tools
Statistical methods

---
