# Supplementary material for: A toolbox of machine learning software to support microbiome analysis
Source: Front Microbiol. 2023 Nov 22;14:1250806. doi: 10.3389/fmicb.2023.1250806 (PMC10704913; doi:10.3389/fmicb.2023.1250806)
Supplement: Supplementary file 3 [file Table_2.DOCX]

**General Software for Machine Learning Applications**

A variety of machine learning software tools are available, with the majority being open source. (Goodswen et al., 2021) have compiled a brief list of ML software tools that are fully or partially intended for machine learning. We have extended this list in Supplementary Table 2 to include additional relevant general software for data analysis and ML applications. These tools are primarily Python and R frameworks that contain collections of software libraries (packages) and require some basic programming knowledge to use. However, some tools like WEKA, KNIME Analytics Platform, and Orange Data Mining, can be used through a GUI without extensive coding or programming expertise to employ popular ML methods.

**Supplementary Table 2.** General Open-source Software and frameworks for Machine Learning and Data Analysis Applications

| **Software Tool Name** | **Main algorithms supported** | **Supported platform** | **Description** | **URL** |
| --- | --- | --- | --- | --- |
| Apache Mahout | Classification, regression, clustering, recommendation engine | Cross-platform | Apache Software Foundation Project for ML algorithms that are mainly focused on linear algebra. | <https://mahout.apache.org/> |
| Caffee | Deep learning | Ubuntu, Red Hat, OS X | Deep learning framework developed by Berkeley AI Research and community contributors. | <http://caffe.berkeleyvision.org/> |
| caret | Classification, regression | Windows, Linux, Mac OS | A powerful machine learning library in R that that contains functions for training and plotting classification and regression models. | <https://cran.r-project.org/web/packages/caret/index.html> |
| CatBoost | Classification, regression | Cross-platform | High-performance open source library for gradient boosting on decision trees with GPU-enabled training. | <https://catboost.ai/> |
| Colab | PyTorch, Keras, TensorFlow and OpenCV libraries are supported | Cloud Service | Google cloud service that supports Python | <https://colab.research.google.com/notebooks/welcome.ipynb> |
| DataExplorer | Data Exploratory Analysis | Windows, Linux, Mac OS | Mainly focuses on exploratory data analysis, feature engineering and data reporting | <https://cran.r-project.org/web/packages/DataExplorer/vignettes/dataexplorer-intro.html> |
| e1071 | Classification, regression, clustering | Windows, Linux, Mac OS | Package that provides various functions for latent class analysis, short time Fourier transform, fuzzy clustering, support vector machines, generalized k-nearest neighbor, bagged clustering, naive Bayes, etc. | <https://cran.r-project.org/web/packages/e1071/index.html> |
| Gephi | Exploratory Data Analysis, Link Analysis, Biological Network Analysis, Visualization of Communities and Clusters in Networks | Windows, Mac OS X, Linux | Gephi is the leading visualization and exploration software for all kinds of graphs and networks. | <https://gephi.org/> |
| glmnet | Lasso and elastic-net regularized generalized linear models that can be used for regression and classification. | Windows, Linux, Mac OS | Package that fits a generalized linear model via penalized maximum likelihood. It is used to implement LASSO and Elastic-Net Regularized Generalized Linear Models | [https://CRAN.R-project.org/package=glmnet](https://cran.r-project.org/package=glmnet) |
| igraph | Network analysis algorithms that can be used for clustering | Windows, Linux, Mac OS | igraph is a library collection for creating and manipulating graphs and analyzing networks. It is written in C and also exists as Python and R packages. | <https://cran.r-project.org/web/packages/plotly/plotly.pdf>  <https://igraph.org/> |
| Keras | Deep learning | Cross-platform | Deep learning framework for Python which runs on top of the TensorFlow platform. | <https://keras.io/> |
| kernLab | Classification, regression, clustering, dimensionality reduction | Windows, Linux, Mac OS | Kernel-based ML lab is a package for classification, regression, clustering, dimensionality reduction etc. | https://cran.r-project.org/web/packages/kernlab/index.html |
| KNIME | Classification, regression, clustering, | Linux, Windows, Mac OS | GUI-based data analytics tool, which also supports integration of the code implemented in Python or R | <https://www.knime.com/> |
| LightGBM | Classification, regression | Linux, Windows, Mac OS | Gradient boosting framework for tree-based ML algorithm that supports parallel, distributed, and GPU Learning. | <https://lightgbm.readthedocs.io/en/v3.3.2/> |
| MICE | Algorithm for imputation of missing values | Linux, Windows, Mac OS | Multivariate Imputation by Chained Equations Package (MICE) provides multiple imputation of missing data using Fully Conditional Specification as outlined in (Buuren and Groothuis-Oudshoorn, 2011) | <https://cran.r-project.org/web/packages/mice/index.html> |
| Microsoft Cognitive Toolkit | Deep learning | Windows, Linux | An open-source toolkit for commercial-grade distributed deep learning | <https://www.microsoft.com/en-us/cognitive-toolkit/> |
| missForest | Missing value imputation | Linux, Windows, Mac OS | missForest is an R package for missing value imputation that uses random forest algorithm. It is based on the paper ‘Nonparametric Missing Value Imputation using Random Forest’ by (Stekhoven and Bühlmann, 2012) | <https://github.com/stekhoven/missForest> |
| MLflow | Machine learning lifecycle management platform. | Open platform that is Python and JavaScript based. | MLflow works in different serving environments, providing REST, Python, R and Java APIs. It is compatible with any deployment tool, or algorithm. MLflow runs the same way in any cloud. | <https://mlflow.org> |
| mlr3 | Machine learning interface for many learning algorithms available on CRAN. | Linux, Windows, Mac OS | Object-oriented and extensible framework for classification, regression, survival analysis, and other machine learning tasks in R (Lang et al., 2019). | <https://mlr3.mlr-org.com/> |
| MXM R Package | Feature selection and Bayesian Networks | Linux, Windows, Mac OS | The MXM R Packageis a collection of utility functions for feature selection, cross validation, and Bayesian Networks. The package supports conditional independence tests for various combinations of target and predictor variables (continuous, categorical). MXM offers many feature selection algorithms that can be used to improve the performance of downstream analysis tasks such as regression and classification, by excluding irrelevant and redundant variables. | https://cran.r-project.org/web/packages/MXM/index.html |
| NetworkX | Network analysis algorithms | Linux, Windows, Mac OS | A Python library for the creation, manipulation, and study of the structure, dynamics, and functions of complex networks | <https://github.com/networkx/networkx> |
| Octave | Classification, regression, clustering, neural networks, deep learning, time series, anomaly detections, statistics | Linux, Windows, MAC OS | Octave is a high-level programming language, with built-in support for numerical linear algebra, compatible with MATLAB, which is useful to prototype ML algorithms. | <https://octave.org/> |
| OpenML | All Machine learning algorithms | Open platform | Open platform for sharing datasets, algorithms, and experiments. | <https://www.openml.org> |
| ORANGE | Classification, regression, clustering | Linux, Windows, Mac OS | GUI-based data analytics tool for the design of ML workflows. New methods can be integrated, developing them as widgets using the Python language. | https://orangedatamining.com/ |
| randomForest | Classification, regression | Linux, Windows, Mac OS | Based on Breiman’s paper on Random forest Classification and regression (Breiman, 2001) | <https://cran.r-project.org/web/packages/randomForest/index.html>  . |
| Rapids | Classification, regression, clustering, dimensionality reduction, time series analysis, graph analysis | Windows, Linux | Data science framework that incorporates multiple open-source libraries and APIs and offers the ability to execute end-to-end ML and analytics pipelines entirely on GPU. | <https://rapids.ai/> |
| rpart | Classification, regression | Linux, Windows, Mac OS | Package used to build classification and regression models by implementing recursive partitioning. | <https://www.rdocumentation.org/packages/rpart/versions/4.1-15> |
| PyCaret | Classification, regression, clustering, anomaly detection, natural language processing, association rule mining, time series forecasting | Linux, Windows, Mac OS | Low-cost Python ML library that automates the workflows and hyperparameter tuning for training and testing models. PyCaret wraps several ML libraries and frameworks, i.e., scikit-learn, XGBoost, LightGBM, CatBoost, etc. | <https://pycaret.org/> |
| PyTorch | Deep learning, | Linux, Windows, Mac OS | ML framework based on the Python programming language and Torch library. | <https://pytorch.org/> |
| Scikit-learn | Classification, regression, clustering, dimensionality reduction | Linux, Windows, Mac OS | Provides a library for ML in Python. | <https://scikit-learn.org/stable/> |
| Shogun | Classification, regression, clustering, dimensionality reduction | Linux, UNIX, Windows, Mac OS | ML toolbox written in C++, interfaces to MATLAB^TM^, R, Octave, Python and also has a stand-alone command line interface. | <https://github.com/shogun-toolbox/shogun> |
| Spark ML | Classification, regression, clustering, collaborative filtering | Cross-platform | A Spark framework. It is an updated version of Spark MLlib API. | <https://spark.apache.org/docs/1.2.2/ml-guide.html> |
| superml | Classification, regression, clustering | Linux, Windows, Mac OS | Provides an interface to build ML models using Python’s Scikit-Learn library in R | <https://cran.r-project.org/web/packages/superml/superml.pdf> |
| TensorFlow | Neural networks | Linux, Windows, Mac OS | Provides Python and JavaScript library for building, training, and deploying ML models in the cloud, browser, on-prem, or on-device. | <https://www.tensorflow.org/> |
| Theano | Deep learning | Cross-platform | Provides a library for deep learning in Python | <http://www.deeplearning.net/software/theano/> |
| Tidymodel | Classificastion, regression, clustering | Linux, Windows, Mac OS | The tidymodels framework is a collection of packages for modeling and machine learning using tidyverse principles | <https://www.tidymodels.org/> |
| WEKA | Classification, regression, clustering, | Linux, Windows, Mac OS | GUI-based tool that contains a collection of ML algorithms for data mining. WEKA can also be called from Java code written by a user. | <https://www.cs.waikato.ac.nz/ml/weka/> |
| WGCN | Weighted Correlation Network Analysis | Windows, Linux, Mac OS | WGCN: Weighted Correlation Network Analysis is an R package for is a comprehensive collection of R functions for performing various aspects of weighted correlation network analysis. The package includes functions for network construction, module detection, gene selection, calculations of topological properties, data simulation, visualization, and interfacing with external software (Zhang and Horvath, 2005). | <https://cran.r-project.org/web/packages/WGCNA/index.html> |
| XGBoost | Classification, regression | Cross-platform | Open source with GPU support library that implements ML algorithms using the Gradient Boosting model. | <https://xgboost.readthedocs.io/en/stable/> |

**References:**

Breiman, L. (2001). Random Forests. *Mach. Learn.* 45, 5–32. doi: 10.1023/A:1010933404324.

Buuren, S. van, and Groothuis-Oudshoorn, K. (2011). mice: Multivariate Imputation by Chained Equations in R. *J. Stat. Softw.* 45, 1–67. doi: 10.18637/jss.v045.i03.

Goodswen, S. J., Barratt, J. L. N., Kennedy, P. J., Kaufer, A., Calarco, L., and Ellis, J. T. (2021). Machine learning and applications in microbiology. *FEMS Microbiol. Rev.* 45, fuab015. doi: 10.1093/femsre/fuab015.

Lang, M., Binder, M., Richter, J., Schratz, P., Pfisterer, F., Coors, S., et al. (2019). mlr3: A modern object-oriented machine learning framework in R. *J. Open Source Softw.* 4, 1903. doi: 10.21105/joss.01903.

Stekhoven, D. J., and Bühlmann, P. (2012). MissForest—non-parametric missing value imputation for mixed-type data. *Bioinformatics* 28, 112–118. doi: 10.1093/bioinformatics/btr597.

Zhang, B., and Horvath, S. (2005). A general framework for weighted gene co-expression network analysis. *Stat. Appl. Genet. Mol. Biol.* 4, Article17. doi: 10.2202/1544-6115.1128.
